# Traumatic Brain Injury in Sports: A Review

**DOI:** 10.1155/2012/659652

**Published:** 2012-07-09

**Authors:** Christopher S. Sahler, Brian D. Greenwald

**Affiliations:** Department of Physical Medicine and Rehabilitation, The Mount Sinai Hospital, One Gustave L. Levy Place, P.O. Box 1240, New York, NY 10029, USA

## Abstract

Traumatic brain injury (TBI) is a clinical diagnosis of neurological dysfunction following head trauma, typically presenting with acute symptoms of some degree of cognitive impairment. There are an estimated 1.7 to 3.8 million TBIs each year in the United States, approximately 10 percent of which are due to sports and recreational activities. Most brain injuries are self-limited with symptom resolution within one week, however, a growing amount of data is now establishing significant sequelae from even minor impacts such as headaches, prolonged cognitive impairments, or even death. Appropriate diagnosis and treatment according to standardized guidelines are crucial when treating athletes who may be subjected to future head trauma, possibly increasing their likelihood of long-term impairments.

## 1. Introduction

Traumatic brain injury has received increased attention, both in the medical literature and social media, particularly in the field of sports. There are 1.7 million documented TBIs annually, with estimates closer to around 3.8 million [1], 173,285 of which are sports- and recreation-related TBIs among children and adolescents [2]. As the number of participants in youth sports continues to grow, the incidence of brain injury is proportionally increasing as well [2]. There is a greater awareness of potential short- and long-term sequelae of athletes who suffer brain injuries such as increased propensity for reinjury, cognitive slowing, early onset Alzheimer's, second impact syndrome, and chronic traumatic encephalopathy [3–23]. Federal and State governments, along with many sport's governing bodies are implementing rule and policy changes designed to increase protection of athletes and to standardize medical care. There is an inherent risk in many sports for repetitive head trauma that athletes subject themselves to and it may be up to the physician to protect their well-being. It is important to understand that athletes are a unique demographic of patients who have many behaviors that may differ from the “normal” office patient.

The evaluation and management of an athlete with TBI includes symptoms assessment, medical examination, and neurocognitive testing with serial evaluations over the following days, weeks, to months of recovery. An initial cognitive and physical rest period followed by a gradual increase in physiologic and cognitive stress in asymptomatic athletes is the hallmark of management and change in the paradigm of management. Proper treatment includes accurate assessment and management using current guidelines in an attempt to minimize potential future deleterious effects from TBI. The purpose of this paper is to provide a review of contemporary views of mild traumatic brain injury in sports including definition, epidemiology, pathophysiology, diagnosis, and management including return to play. The timeliness of this paper is apparent now that 37 States have established laws requiring youths who sustain a sporting related brain injury be required to see a physician prior to returning to play; as of August 2011, to the best of the authors' knowledge. Schools, communities, and athletic leagues must be aware of these legislations and follow them appropriately.

## 2. Materials and Methods

Key articles from major sources that are considered the gold standard of knowledge within this topic were reviewed to give a comprehensive up-to-date review of this topic. Pubmed was used to search and identify supplementary articles for supporting data and topics. There were sources and articles used in this paper including epidemiology were found using the Centers for Disease Control and Prevention (CDC) website on traumatic brain injury.

## 3. Definition

The term mild TBI (mTBI) is now used in place of concussion in the nomenclature according to the Centers for Disease Control and Prevention CDC and the World Health Organization (WHO). Traumatic brain injury is a clinical diagnosis of neurological dysfunction following head trauma. Multiple definitions exist however the CDC defines a mTBI as a complex pathophysiologic process affecting the brain, induced by traumatic biomechanical forces secondary to direct or indirect forces to the head. The American Academy of Neurology (AAN) defines mTBI as a biomechanically induced brain injury resulting in neurologic dysfunction [24]. MTBI results in a constellation of physical, cognitive, emotional, and/or sleep-related symptoms and may or may not involve a loss of consciousness (LOC). Duration of symptoms is highly variable and may last from several minutes to days, weeks, months, or even longer in some cases [25]. There is inherent weakness in all the definitions of mTBI as they are based on clinical evaluation and may be biased by the examiner or examinee.

## 4. Epidemiology

According to the CDC, there are 1.7 million documented TBIs each year, with estimates closer to around 3.8 million [1]. Direct medical costs and indirect costs such as lost productivity of TBI totaled an estimated $76.5 billion in the United States in 2000 [26, 27]. Annually, US emergency departments (EDs) treat an estimated 173,285 sports- and recreation-related TBIs among children and adolescents, ages ranging from birth to 19 years averaged over 10 years [2]. During the same 10-year period, ED visits for sports- and recreation-related mTBIs among children and adolescents increased by 60% annually [2], from 153,375 to 248,418 in 2009 [28]. ED visits for mTBI occurring in organized team sports almost doubled in children aged 8 to 13 years and more than tripled among youths aged 14 to 19 years from 1997 to 2007 [29]. Breakdown of these numbers show that 71.0% of all sports- and recreation-related TBI emergency department visits were males and 70.5% of total visits were among persons aged 10–19 years [2]. Overall, the activities most commonly associated with TBI-related ED visits included bicycling and football; followed by playground activities, basketball, and soccer [2, 28]. 

CDC data is not available for non-ED visits which include primary care and specialist office visits because the collection of brain injury data is easier to collect and quantify in ED patients. Increase incidence is multifactorial, and is in part due to the increase in participation of our youth in athletic activities [31]. There is also increased awareness by the general public including parents and coaches to report and refer patients with concussive symptoms for physician evaluation. 

## 5. Pathophysiology

TBIs are a result of dysfunction in neuronal metabolism and the microscopic anatomy of the brain that occurs in two distinct phases. Diffuse axonal injury (DAI) is the hallmark injury of TBI and occurs during an initial phase of neuronal and parenchymal as the direct result of the traumatic force. DAI is a result of a rotational forces, and is important to distinguished from cortical contusions or other hemorrhages due to linear acceleration/deceleration injury [32]. A secondary delayed phase of the brain injury model includes inflammatory cascade activation, edema, ischemia, effects of free radicals, excitatory amino acids, ion release, and programmed cell death [33]. Disruption of axonal neurofilament organization occurs and impairs axonal transport leading to axonal swelling, Wallerian degeneration, and transection [34]. Release of excitatory neurotransmitters acetylcholine, glutamate and aspartate, and the generation of free radicals may also contribute to secondary injury [35].

## 6. Clinical Presentation of TBI in Athletes

The clinical signs and symptoms of mTBIs may range from subtle mood changes to obvious loss of consciousness. The onset of symptoms may be immediately following the injury, or several minutes later [36]. The AAN identifies signs of mTBI to be amnesia, behavior or personality changes, confabulation, delayed verbal and motor responses, disequilibrium, orientation, emotional labiality, loss of consciousness, slurred/incoherent speech, or a vacant stare. Symptoms of mTBI may include blurry/double vision, confusion, dizziness, excessive drowsiness, sleep difficulties, feeling hazy, foggy, or groggy, headache, inability to focus or concentrate, nausea, vomiting, and photo- or phonophobia [24]. Mood changes, emotional outburst, and behavioral changes also may be the principle manifesting symptoms of mTBI. Mild TBI should also be only a part of a broader differential diagnosis of the previously mentioned signs and symptoms of other common sports-related conditions such as poorly fitting helmet, dehydration, migraine headache, heat exhaustion/stroke, metabolic disturbances, and cardiac or other medical conditions.

Clinicians need consider athletes a unique population subset during evaluations. The sporting world has a culture and mentality that is predicated on pushing athletes beyond their perceived physical and mental abilities. This includes participating in adverse conditions and through a multitude of injuries. Athlete's desire to better themselves and help their team succeed will frequently supersede all other considerations, even at risk to their own bodily harm. Athletes are well known to underreport symptoms that may exclude them from participation. Long standing philosophies such as “getting their bell rung” is often just an accepted part of athletic competition. Other considerations are signs or symptoms of TBI may only be presents under stressful, high exertion game-like conditions. There may also be other incentives and outside motivators to perform well in athletic arena such as the presence of professional scouts, possible scholarships, advancement to a higher-level team, or even money. Coaches may not fully disclose all information attempting to keep key players on the playing field. Parents who desire to see their children perform well may not wish to have their son or daughter pulled from the sporting event. These unique circumstances and conditions must be taken into account by physicians when evaluating athletes.

## 7. On the Field Assessment and Management

Sports-related mTBI is a common and challenging injury to diagnose, with a constellation of signs and symptoms that can evolve over hours or days after a concussive episode. Evaluation of mTBI should begin with cervical spine evaluation given the similar mechanism of action in both processes. It is important to note that players who sustain severe head trauma causing a loss of consciousness require prompt, on the field assessment of airway, breathing, circulation, and immediate stabilization of the neck with helmet and shoulder pads left on. Those athletes who have persistent loss of consciousness (LOC) or alteration of consciousness should be kept in a stable position and rapidly transported on a backboard and ambulance to an emergency room. However, most athletes will not suffer LOC and may be evaluated on the sidelines.

Any player suspected of sustaining a mTBI should be immediately removed from the playing field for proper evaluation. If a player has a suspected brain injury and a physician is not present at the venue, the player must be removed from practice or play and referred for proper evaluation before being able to return to play. The point should be made again that most concussions to do involve loss of consciousness. There is also the possibility of delayed symptoms or neurologic decline in these patients, which makes it imperative to perform serial examinations. Multiple studies have shown that collegiate and high school level athletes may demonstrate delayed onset of neuropsychological deficits and symptoms post-injury [37–42]. If the diagnosis of TBI is made, the athlete is required to sit out the remainder of the game or competition. Initial treatment should begin with symptomatic management by reducing the physical and cognitive stressors that may be profound in the sporting arena. The bright lights and loud noises should be minimized which may require removing the athlete completely from the sporting complex. The player initially may require mild analgesics for persistent headache for which Tylenol or NSAIDs may be prescribed. Relative cognitive and complete physical rest should be maintained for at least 24 hours or until follow up evaluation with a physician can be made to begin the return to play protocol.

The paradigm shift in recent years has moved the focus of the initial assessment from grading the severity of the TBI to injury detection and characterization [43]. The scales previously used for grading TBIs have been the Cantu and Colorado guidelines. These guidelines stratify the severity of the TBI based on presence/duration of loss of consciousness (LOC) and presence/duration of amnesia or confusion. Management of that athlete and the RTP is then based on the grade of mTBI they receive at the time of initial assessment, however, this is no longer the current recommended practice.

The primary assessment used today by sports medicine physicians is the SCAT-2 (Sport Concussion Assessment Tool-2), which is a product of the consensus guidelines established in Zurich in 2008 during the 3rd International Conference on Concussion in Sport. Although no prospective studies exist establishing its efficacy, it is believed to be the best screening tool as it incorporates the key components from other scales, and was constructed by the leaders in the field of mTBI in sports in the form of consensus guidelines. Components include review of subjective symptoms, the Glasgow coma scale, the standardized assessment of concussion (SAC) cognitive assessment, Maddocks score, and an evaluation of balance and coordination. Scores of the SCAT-2 can be summated, however clinicians should be mindful that there is not a “normal score” or score cut off to allow RTP (Supplementary material available online at doi:10.1155/2012/659652). The SCAT-2 is most effective when it is compared to a baseline screen, as well as serial examinations following a TBI. Athletes seen in the office setting undergo detailed evaluation including history and past medical history, neurologic examination focusing on coordination and balance, and cognitive functioning. 

In addition to having properly trained medical professionals performing TBI assessments, it is important to ensure coaches, trainers, players, and family are also educated about the possible signs and symptoms to ensure early recognition. Physicians are not present at all the athletic venues in which TBIs may occur such as practices or training sessions. The CDC has an initiative termed “Heads Up” to educate not only physicians, but also coaches, parents, schools, and athletes on preventing, recognizing, and responding to TBIs. Information includes statistics, fundamentals of TBI, sign and symptom lists, prevention techniques, and treatment protocols with wording that is directed for their respective audience. Also available are pocket size cards with condensed information on recognition, assessment, and management that is available for non-medical professionals to take out in the field. Studies have found that the coaches' version of the toolkit helped them to better identify signs and symptoms of mTBI, increased their awareness of the requirement of health care professional evaluation, and provided helpful information about possible length of recovery [44]. Chrisman et al found physicians were more likely to be aware of and to follow recommended guidelines for RTP activity after reading the Heads Up toolkit than those who did not [45]. 

## 8. Neurocognitive Evaluations and the Role of Baseline Testing

Available baseline cognitive screening tools include neurocognitive testing, Immediate Measurement of Performance and Cognitive Testing (ImPACT), Brain Injury Screening Questionnaire (BISQ), Automated Neuropsychological Assessment Metrics (ANAM), CogSport (formerly Concussion Sentinel), Concussion Resolution Index (CRI), and the Standardized Assessment of Concussion (SAC). Evaluation of many scales including the SCAT-PCSS, IMPACT-PCSS, Signs and Symptoms checklist, Pittsburgh Steelers Post Concussion Scale, Concussion Symptom Inventory, and the Head Injury Scale did not find one particular scale statistically superior to the rest in screening for TBI, however, neurocognitive evaluation was not included [43]. Computerized and traditional neurocognitive testing of verbal and visual memory, complex attention, reaction time, and processing speed is a useful tool to diagnose and to track athletes when baseline testing is performed and compared with post-injury scores [46]. The Zurich consensus guidelines state that neurocognitive testing is the cornerstone to TBI identification and management [47]. Resolution of post-concussive symptoms and return to baseline cognitive status typically are thought to occur on similar timelines [48]. However, when comparison of baseline and post-injury results in a group of collegiate athletes, 83% of athletes with concussions had significantly lower neurocognitive test scores when compared with their baseline scores demonstrating that neurocognitive testing was nearly 20% more sensitive for detecting injury than symptom reporting alone [49]. None of the athletes in the control group had symptoms or lower scores on neurocognitive testing demonstrating a high sensitivity and specificity for neurocognitive testing in identifying concussion. Similar studies have also confirmed these findings demonstrating the “added value” of computerized neurocognitive testing [50]. This clearly identifies the integral role of neurocognitive testing in the management of TBI in the athletic venue. Administration of traditional neuro-psychologic testing to this point has not been available for all athletes mainly due to the financial cost and resources required to administer the examination, specifically a trained neuropsychologist. One solution is through the use of computerized testing which presents many advantages. Computerized neurocognitive testing has been shown to provide sensitive and specific objective data to quantify injury and track recovery [49–51]. Advantages include screening of athletes at a lower financial cost and with only minimal human resource. Also, preseason testing of large numbers of athletes can be now be quickly and efficiently accomplished at most levels of competition. Large databases of information may also be constructed allowing researchers more data for analysis to continue advancing our knowledge in the management of TBI in sports.

Baseline neurocognitive testing is recommended when possible. Cognitive function should be evaluated and tracked following a TBI in an athlete and used as a component in the decision-making management of that player, but never as a sole factor.

## 9. Neuroimaging

Urgent Neurologic imaging does not play a primary role in evaluation or management of an athlete who has sustained a TBI, but is used to rule out significant structural pathology such as intracerebral hemorrhage. Prolonged unconsciousness, persistent mental status alterations, or abnormalities on neurologic examination require urgent neuroimaging [24]. Other commonly used criteria for urgent head computerized tomography (CT) scan in the acute setting include the Canadian CT head rule which require a CT scan if concussed patients have any one of the following: GCS <15 two hours after injury, suspected open or depressed skull fracture, any sign of basilar skull fracture (hemotympanum, periorbital bruising or raccoon eyes, retroauricular bruising or battle's sign, cerebrospinal fluid leak, oto- or rhinorrhea), two or more episodes of vomiting, 65 years of age or older, amnesia before impact of 30 or more minutes, or dangerous mechanism (pedestrian struck by motor vehicle, occupant ejected from motor vehicle, fall from =3 feet or =5 stairs) [52]. Using this criteria has a 100 percent sensitivity and 88 to 40 percent specificity in detecting neurosurgical and clinically important brain injury abnormalities [53, 54]. Imaging may also be considered in patients who have worsening symptoms, severe acute headaches, or failure of resolution of symptoms within a few weeks. Alternative imaging modalities such as MR (sequences including gradient echo, perfusion, diffusion tensor imaging), functional MRI, and PET scans are all informative into the pathophysiology of TBI, but are not currently recommended as a component of clinical management [47]. Limitations of these modalities include financial cost, limited equipment availability, and lack of evidence guiding changes in management. Future work also includes the use of transcranial Doppler to evaluate cerebrovascular reactivity abnormalities in TBI. Asymptomatic TBI patients at rest had physiologically stress-induced impairments of cerebrovascular reactivity when compared to control subjects representing brains which have not fully healed [55]. In the future, addition cerebrovascular flow studies would add another component to determine whether athletes have recovered physiologically prior to returning to play.

## 10. Return to Play Criteria

The key feature of TBI management in sports is physical and cognitive rest until symptoms resolve. A graduating program of exertion and cognitive workload prior to medical clearance and return to play. The basis behind the RTP criteria is that a concussed brain has a lower threshold of reinjury in the first few days or weeks following the initial injury [56]. Recovery times may be longer in adolescents and children [57]. An athlete who returns to play within this vulnerable time period risks permanent disability or even death [58, 59]. Athletes are unique in particular regarding the desire to quickly return to the same venue in which the brain injury was sustained. The RTP guidelines are established to protect the health of the athletes.

Previously, the Cantu and Colorado guidelines were used basing the RTP criteria on severity of mTBI and number of mTBIs that season. Although they take into account the athletes symptoms when returning to play, there were not established guidelines for a graduating stepwise addition of physical and cognitive workload prior to return to play. The current standard of care is based on the consensus guidelines established at the 3rd International Conference on Concussion in Sport in 2008 when determining RTP (Table 1). The guidelines allow for an initial phase of physical and cognitive rest, with slow reintroduction of physical and cognitive activity in a stepwise fashion, providing the patient remains asymptomatic at each step. There are 6 phases in the protocol starting with complete physical and cognitive rest, then advancing to light aerobic exercise, sport-specific exercise, noncontact training drills, full contact practice, and finally RTP. The initial rest period should not only include complete physical rest, but the athlete's academic work also requires modification. This may include, but is not limited to, a reduced number of work assignments, more time to complete class work and tests, breaking down complex tasks into simple steps, and providing a distraction free area for work. A comprehensive medical examination, incorporating SCAT-2, as well as computerized neurocognitive testing should also be conducted at this point. If the athlete is asymptomatic, they may advance to light aerobic exercise (e.g., walking, swimming, stationary cycle) and may continue to progress through the protocol if they remain asymptomatic. If the athlete becomes symptomatic at any point, they must return to the previous level of activity until symptoms resolve for at least 24 hours.

Unrestricted return to play is permitted when the athlete has progressed through the protocol, is asymptomatic, and has returned to baseline or normal values on neurocognitive testing. Given that 90% of mTBI symptoms resolve within one week [41], this protocol can usually be completed in one week as the athlete advances each step in 24 hours. It is recommended to take a more conservative approach to children and adolescents when evaluating for RTP due to particular risks of this age group (i.e., diffuse cerebral swelling) [47]. The guidelines recommend allowing for an extended amount of time of asymptomatic rest and/or the length of graduated exertion in this population. High school athletes had prolonged impairments on neurocognitive testing when compared to professional football players [60] or collegiate athletes [61, 62]. There is evidence that adult brains may be less susceptible to mTBIs and may be able to RTP sooner. Pelman et al. states that some professional American football players are able to RTP more quickly, with even same day RTP supported by National Football League studies without a risk of recurrence or sequelae [63]. 

It is an important consideration in advancing athletes through the RTP protocol that they remain symptom-free without the use of any pharmacological agents/medications that may mask or modify the symptoms of mTBI [11]. The Zurich consensus guidelines also list modifying factors that require special consideration for RTP criteria and obtaining additional testing such as neuroimaging (Table 2). These include prolonged duration of symptoms, prolonged LOC, seizures, multiple mTBIs especially in the recent past, or change in mental health. Currently, there are no recommendations on the total number of TBIs that are “allowable” for an athlete to sustain before recommending them to sit out the remainder of the season or retiring from the sport. Elite athletes are also recommended to follow the same treatment plan and RTP protocol.

The goal of the guideline is to allow full physical, cognitive, and metabolic recovery to the concussed brain before subjecting it to forces that may cause reinjury. Additional brain trauma within the metabolic recovery window may have both potential short-term and long-term consequences. Even when neuropsychological testing is normal, physiologic, and metabolic dysfunction still may persist for some time. Currently, there are no recommended laboratory testing or imaging modalities that are readily available and reliable to evaluate and follow the microcellular dysfunction. In the future, additional tools may be added into the RTP guidelines such as the previously discussed transcranial Doppler to determine full physiologic recovery.

The evaluation and management of an athlete with TBI is multifactorial assessing symptoms, medical examination, and neurocognitive testing ensuring to catch the variability in presentations of injured athletes. Initial, followed by serial medical and neurocognitive examinations as the patient progresses through the RTP protocol is warranted. Athletes are required to remain fully asymptomatic and returned to baseline cognitive functioning before returning to their sport.

## 11. Sequelae of TBI in Sports

The increase in media attention, legislation, and constant revision of medical guidelines with respect to TBI is due to the increased awareness of short- and long-term consequences. The obvious immediate impact on the athlete is dealing with the symptoms of a TBI including most commonly headaches, but also poor sleep, excessive drowsiness, poor concentration, and poorer cognitive aptitude. It is estimated that 1.8 million individuals develop acute PTHA each year and 400,000 individuals develop chronic PTHA [64]. Considering most athletes are student-athletes, these symptoms will have an obvious impact on their academic performances as well. In studies of high school and collegiate athletes with a history of three or more concussions had a more severe presentation of concussion, [13] were more likely to have baseline headaches [21], were more vulnerable to brain injury than those without concussion history [4], and were three times more likely to sustain an additional injury [65]. Also, repeated mild TBIs occurring within a short period of time (i.e., hours, days, or weeks) may be catastrophic or fatal [3]. 

A growing body of evidence exists linking brain injuries of all severity with long-term sequelae.

Repeated mild TBIs occurring over an extended period of time (i.e., months, years) may result in cumulative neurological and cognitive deficits. Retired American professional football players with a history of three or more TBIs were 5 times more likely to have mild cognitive impairment [12]. Professional boxers are well known to have a risk of significant cognitive decline and alterations in brain function [7]. However, there is increasing concern that cumulative effects may also be occurring in athletes who sustain more “routine” injuries as a function of playing a contact sport such as football or ice hockey [8, 9]. Long term effects of repeated concussions include chronic motor and neuropsychological deficits [10, 11]. Collins et al. found that among 400 collegiate football players with two or more previous TBIs independently predicted long-term deficits of executive function, processing speed, and self-reported symptom severity [8]. The nature, burden, and duration of the clinical postconcussive symptoms may be more important than the presence or duration of amnesia alone [14–16]. A telephone-based survey performed by the University of Michigan Institute of Social Research in association of the National Football League of 1,063 retired NFL players found a 19-fold increase rate of memory-related diseases such as Alzheimer's in the 35–49-year-old age group and a 5-year-old increase in ages 50+ when compared to national control groups. Chronic Traumatic Encephalopathy is an entity classically described in former boxers [20, 66], however, there are increasing numbers of case reports described in the literature of athletes in other sports who have a significant history of TBIs [17]. McKee et al. reviewed the autopsy findings of three professional athletes in addition to published reports of 48 cases of suspected CTE and concluded that it is a neuropathologically distinct, slowly progressive tauopathy with a clear environmental etiology [19]. A full discussion is not within the scope of this paper, however, the point should be understood that an association between CTE and TBI is evident within the literature and warrants consideration and future study.

## 12. Second Impact Syndrome

Second-impact syndrome (SIS) is a rare form of reinjury that occurs prior to the complete resolution of a previous TBI [5]. SIS may result in serious permanent neurologic injury or even death, even if the second impact is only considered to be a minor force. According to the AAN, SIS is a diffuse cerebral dysregulation leading to massive cerebral edema and subsequent herniation. Typically athletes diagnosed with SIS are children or adolescents rather than adults.

Fourteen of the 17 case reports of SIS have occurred in persons less than 20 years old, the others were in a 21 y/o and two 24 years old [6]. This is due to the physiologic differences of children and adolescents compared to adults who have prolonged and diffuse cerebral edema after traumatic brain injury with increased sensitivity to glutamate, increasing their risk to secondary injury [22, 23]. Although rare, SIS has a high associated morbidity and mortality and therefore must be considered.

## 13. Clinical Training of Clinicians in Sports

As important as having a physician conduct the appropriate brain injury evaluation of an athlete is ensuring the appropriate training of that medical professional conducting the examination. Many studies have concluded that most physicians have little to no knowledge on the accurate diagnosis or management of patients with TBI. Powel et al. found in their study that over 50 percent of patients who presented to the emergency department with TBIs were not accurately identified by ED physicians [67]. Surveys to determine the knowledge of TBI guidelines in primary care physicians found that less than half were up to date with current medical management [45]. Of patients admitted to the hospital for TBI, 9% were allowed to RTP too quickly and 60% were given no advice in regards to RTP [68]. In a survey of the members of the American Society of Sports Medicine, only 30% of physicians treated their patients per the current established guidelines [69]. As the incidence of brain injuries continue to increase, there must also be a concurrent increase and improvement of physician knowledge and training regarding assessment and management of TBI in sports.

## 14. Prevention of TBI in Sports

Prevention of TBI is paramount and should be the focus of sporting governing bodies, the athletes, coaching staff, and medical professionals. Two main avenues to accomplish this are through improved protective equipment and rule changes. It has long been understood with literature dating back to the 1960s that hard helmets in sports reduce the incidence of skull fractures and bony head trauma, however, they do not reduce the risk of brain injury [70]. Biomechanical studies which show a reduction in impact forces to the brain with the use of head gear and helmets, but these findings have not been translated to show a reduction in TBI incidence [47]. The use of helmets has been argued to increase brain injury rates through behavioral changes in the athletes who are able to assume a more dangerous playing style and use their helmet as a “weapon” when contacting another player [71]. Clinical evidence that current available protective equipment helps to prevent TBI is not established. Helmets protect against head and facial injury and hence should be recommended for participants in alpine sports [72]. This failure to reduce mTBIs is a product of the biomechanical forces needed to generate the primary neuronal pathology in TBI, diffuse axonal injury (DAI). Helmets are primarily designed to reduce linear accelerative/decelerative forces, not the rotational forces which cause the DAI and in fact may increase rotation forces experienced. Mouth guards have a definite role in preventing dental and oro-facial injuries [47].

The primary means in which rates of TBI incidence in sports will reduce is through rule changes to minimize head impacts moving forward. Penalizing, fining, or suspending athletes who intentionally impact another players head are means to discourage brain trauma. No longer allowing football (soccer) players to head the ball removes a large risk factor as it has been shown that heading accounts for around 50% of brain injuries in sport [73]. 

## 15. Discussion

Traumatic brain injury continues to be a popular topic in the medical community and social media, especially in youth sports. We have seen double to triple the number of ED visits by children and adolescents for evaluation of sports related TBI in the past ten to fifteen years [74]. It is important to understand that athlete should be considered a unique population with its own culture and risk factors. Maximizing performance is often the primary objective, even if that is at the cost of bodily harm. The prior thinking that mTBI only occurred in contact sports is not correct as demonstrated by the incidence of brain injury in soccer players [2, 28, 30, 73]. Cognitive impairments as well as long-term consequences such as early dementia have been linked to recurrent mTBIs and even repetitive subconcussive impacts [8, 9]. Chronic traumatic encephalopathy or second impact syndrome are seen mostly in children and adolescents and are rare but devastating potential sequelae of repetitive brain injuries as well. Protecting athletes using our current understanding of the brain injury model is the primary goal of the medical community serving this group. Under the advisement of the medical community, legislatures have passed laws mandating that youths who are suspected of sustaining a TBI during a game or practice, must be removed from competition and sat out until cleared for RTP by a physician. It is imperative that these athletes are evaluated by a physician trained in sports medicine and familiar with the culture of this subset population. Consensus statement guidelines establish clear management of athletes with TBI and have been outlined in this paper. The evaluation of an athlete with TBI is multifactorial assessing symptoms, medical examination, and neurocognitive testing followed by serial evaluations over the days, weeks, to months of recovery. An initial cognitive and physical rest period, followed by a gradual increase in physiologic and cognitive stress in asymptomatic athletes is the hallmark of management. Athletes are not permitted to return to play until asymptomatic under physiologic stress. Continued education of the general public who may interact with athletes is essential to correctly identify concussed individuals and direct them to appropriate medical care.

Future work should focus on providing evidence to support using the SCAT2 assessment format. Continuing work to improve imaging modalities such as the discussed transcranial Doppler or using serum biomarkers as means to assess and follow physiologic dysfunction and recovery would be excellent additional tools for managing athletes with brain injuries. A better understanding of what and why there appear to be differences in brain injuries in adults compared to children or adolescents and how that would affect RTP management. Presently, the focus should continue to prioritize proper assessment and management by medical professionals based on the current guidelines reviewed in this article, and continued rule changes to minimize head trauma and incidence of brain injury.

## Supplementary Material

Graduated return to play protocol established at the 3rd international conference on concussion in sport.

## Figures and Tables

**Table 1:** Graduated return to play protocol established at the 3rd international conference on concussion in sport.

Rehabilitation state	Functional exercise at each stage of rehabilitation	Objective of each stage
(1) No activity	Complete physical and cognitive rest	Recovery
(2) Light aerobic exercise	Walking, swimming, or stationary cycle keeping intensity <70% MPHR; no resistance training	Increase HR
(3) Sport-specific exercise	Skating drills in ice hockey, running drills in soccer, no head impact activates	Add movement
(4) Non-contact training drills	Progression to more complex training drills, for example, passing drills in football and ice hockey, may start progressive resistance training	Exercise, coordination, and cognitive load
(5) Full contact practice	Following medical clearance; participate in normal training activates	Restore confidence and asses functional skills by coaching staff
(6) Return to play	Normal game play	

**Table 2:** Modifying factors in concussion management from consensus guidelines.

Factors	Modifier
	Number
symptoms	Duration (>10 days)
	Severity
Signs	Prolonged loss of consciousness (.1 min), amnesia
Sequelae	Concussive convulsions
	Frequency—repeated concussions over time
Temporal	Timing—injuries close together in time
	“Recency”—recent concussion or traumatic brain injury
Threshold	Repeated concussions occurring with progressively less impact force or slower recovery after each successive concussion
Age	Child and adolescent (18 years old)
Co- and premorbidities	Migraine, depression or other mental health disorders, attention deficit hyperactivity disorder, learning disabilities, sleep disorders
Medication	Psychoactive drugs, anticoagulants
Behavior	Dangerous style of play
Sport	High risk activity, contact and collision sport, high sporting level
