# Supplementary material for: Traumatic Brain Injury in Sports: A Review
Source: Rehabil Res Pract. 2012 Jul 9;2012:659652. doi: 10.1155/2012/659652 (PMC3400421; doi:10.1155/2012/659652)
Supplement: Supplementary file 1 — Graduated return to play protocol established at the 3rd international conference on concussion in sport. [file 659652.f1.pdf]

SCAT2

Sport Concussion Assessment Tool 2

Name ______________________

Sport/team ______________________

Date/time of injury ______________________

Date/time of assessment ______________________

Age ____________ Gender ☐ M ☐ F

Years of education completed ______________________

Examiner ______________________

What is the SCAT2?[1]

This tool represents a standardized method of evaluating injured athletes for concussion and can be used in athletes aged from 10 years and older. It supersedes the original SCAT published in 2005[2]. This tool also enables the calculation of the Standardized Assessment of Concussion (SAC)[3,4] score and the Maddocks questions[5] for sideline concussion assessment.

Instructions for using the SCAT2

The SCAT2 is designed for the use of medical and health professionals. Preseason baseline testing with the SCAT2 can be helpful for interpreting post-injury test scores. Words in Italics throughout the SCAT2 are the instructions given to the athlete by the tester.

This tool may be freely copied for distribtion to individuals, teams, groups and organizations.

What is a concussion?

A concussion is a disturbance in brain function caused by a direct or indirect force to the head. It results in a variety of non-specific symptoms (like those listed below) and often does not involve loss of consciousness. Concussion should be suspected in the presence of **any one or more** of the following:

- Symptoms (such as headache), or
- Physical signs (such as unsteadiness), or
- Impaired brain function (e.g. confusion) or
- Abnormal behaviour.

Any athlete with a suspected concussion should be REMOVED FROM PLAY, medically assessed, monitored for deterioration (i.e., should not be left alone) and should not drive a motor vehicle.

Symptom Evaluation

How do you feel?

You should score yourself on the following symptoms, based on how you feel now.

	none	mild		moderate		severe	
Headache	0	1	2	3	4	5	6
"Pressure in head"	0	1	2	3	4	5	6
Neck Pain	0	1	2	3	4	5	6
Nausea or vomiting	0	1	2	3	4	5	6
Dizziness	0	1	2	3	4	5	6
Blurred vision	0	1	2	3	4	5	6
Balance problems	0	1	2	3	4	5	6
Sensitivity to light	0	1	2	3	4	5	6
Sensitivity to noise	0	1	2	3	4	5	6
Feeling slowed down	0	1	2	3	4	5	6
Feeling like "in a fog"	0	1	2	3	4	5	6
"Don't feel right"	0	1	2	3	4	5	6
Difficulty concentrating	0	1	2	3	4	5	6
Difficulty remembering	0	1	2	3	4	5	6
Fatigue or low energy	0	1	2	3	4	5	6
Confusion	0	1	2	3	4	5	6
Drowsiness	0	1	2	3	4	5	6
Trouble falling asleep (if applicable)	0	1	2	3	4	5	6
More emotional	0	1	2	3	4	5	6
Irritability	0	1	2	3	4	5	6
Sadness	0	1	2	3	4	5	6
Nervous or Anxious	0	1	2	3	4	5	6

Total number of symptoms (Maximum possible 22) ☐

Symptom severity score ☐

(Add all scores in table, maximum possible: 22 x 6 = 132)

Do the symptoms get worse with physical activity? ☐ Y ☐ N

Do the symptoms get worse with mental activity? ☐ Y ☐ N

Overall rating

If you know the athlete well prior to the injury, how different is the athlete acting compared to his / her usual self? Please circle one response.

no different	very different	unsure

Cognitive & Physical Evaluation

1 Symptom score (from page 1)

22 **minus** number of symptoms — of 22

2 Physical signs score

Was there loss of consciousness or unresponsiveness? Y N

If yes, how long? minutes

Was there a balance problem/unsteadiness? Y N

Physical signs score (1 point for each negative response) — of 2

3 Glasgow coma scale (GCS)

Best eye response (E)

No eye opening	1
Eye opening in response to pain	2
Eye opening to speech	3
Eyes opening spontaneously	4

Best verbal response (V)

No verbal response	1
Incomprehensible sounds	2
Inappropriate words	3
Confused	4
Oriented	5

Best motor response (M)

No motor response	1
Extension to pain	2
Abnormal flexion to pain	3
Flexion/Withdrawal to pain	4
Localizes to pain	5
Obeys commands	6

Glasgow Coma score (E + V + M) — of 15

GCS should be recorded for all athletes in case of subsequent deterioration.

4 Sideline Assessment – Maddocks Score

"I am going to ask you a few questions, please listen carefully and give your best effort."

Modified Maddocks questions (1 point for each correct answer)

At what venue are we at today?	0	1
Which half is it now?	0	1
Who scored last in this match?	0	1
What team did you play last week/game?	0	1
Did your team win the last game?	0	1

Maddocks score — of 5

Maddocks score is validated for sideline diagnosis of concussion only and is not included in SCAT 2 summary score for serial testing.

5 Cognitive assessment

Standardized Assessment of Concussion (SAC)

Orientation (1 point for each correct answer)

What month is it?	0	1
What is the date today?	0	1
What is the day of the week?	0	1
What year is it?	0	1
What time is it right now? (within 1 hour)	0	1

Orientation score — of 5

Immediate memory

"I am going to test your memory. I will read you a list of words and when I am done, repeat back as many words as you can remember, in any order."

Trials 2 & 3:

"I am going to repeat the same list again. Repeat back as many words as you can remember in any order, even if you said the word before."

Complete all 3 trials regardless of score on trial 1 & 2. Read the words at a rate of one per second. Score 1 pt. for each correct response. Total score equals sum across all 3 trials. Do not inform the athlete that delayed recall will be tested.

List	Trial 1		Trial 2		Trial 3		Alternative word list		
elbow	0	1	0	1	0	1	candle	baby	finger
apple	0	1	0	1	0	1	paper	monkey	penny
carpet	0	1	0	1	0	1	sugar	perfume	blanket
saddle	0	1	0	1	0	1	sandwich	sunset	lemon
bubble	0	1	0	1	0	1	wagon	iron	insect
**Total**									

Immediate memory score — of 15

Concentration

Digits Backward:

"I am going to read you a string of numbers and when I am done, you repeat them back to me backwards, in reverse order of how I read them to you. For example, if I say 7-1-9, you would say 9-1-7."

If correct, go to next string length. If incorrect, read trial 2. One point possible for each string length. Stop after incorrect on both trials. The digits should be read at the rate of one per second.

			Alternative digit lists		
4-9-3	0	1	6-2-9	5-2-6	4-1-5
3-8-1-4	0	1	3-2-7-9	1-7-9-5	4-9-6-8
6-2-9-7-1	0	1	1-5-2-8-6	3-8-5-2-7	6-1-8-4-3
7-1-8-4-6-2	0	1	5-3-9-1-4-8	8-3-1-9-6-4	7-2-4-8-5-6

Months in Reverse Order:

"Now tell me the months of the year in reverse order. Start with the last month and go backward. So you'll say December, November ... Go ahead"

1 pt. for entire sequence correct

Dec-Nov-Oct-Sept-Aug-Jul-Jun-May-Apr-Mar-Feb-Jan — 0 1

Concentration score — of 5

[1] This tool has been developed by a group of international experts at the 3rd International Consensus meeting on Concussion in Sport held in Zurich, Switzerland in November 2008. The full details of the conference outcomes and the authors of the tool are published in British Journal of Sports Medicine, 2009, volume 43, supplement 1.
The outcome paper will also be simultaneously co-published in the May 2009 issues of Clinical Journal of Sports Medicine, Physical Medicine & Rehabilitation, Journal of Athletic Training, Journal of Clinical Neuroscience, Journal of Science & Medicine in Sport, Neurosurgery, Scandinavian Journal of Science & Medicine in Sport and the Journal of Clinical Sports Medicine.

[2] McCrory P et al. Summary and agreement statement of the 2nd International Conference on Concussion in Sport, Prague 2004. British Journal of Sports Medicine. 2005; 39: 196-204

[3] McCrea M. Standardized mental status testing of acute concussion. Clinical Journal of Sports Medicine. 2001; 11: 176-181

[4] McCrea M, Randolph C, Kelly J. Standardized Assessment of Concussion: Manual for administration, scoring and interpretation. Waukesha, Wisconsin, USA.

[5] Maddocks, DL; Dicker, GD; Saling, MM. The assessment of orientation following concussion in athletes. Clin J Sport Med. 1995;5(1):32–3

[6] Guskiewicz KM. Assessment of postural stability following sport-related concussion. Current Sports Medicine Reports. 2003; 2: 24-30

6 Balance examination

This balance testing is based on a modified version of the Balance Error Scoring System (BESS)[6]. A stopwatch or watch with a second hand is required for this testing.

Balance testing

"I am now going to test your balance. Please take your shoes off, roll up your pant legs above ankle (if applicable), and remove any ankle taping (if applicable). This test will consist of three twenty second tests with different stances."

(a) Double leg stance:

"The first stance is standing with your feet together with your hands on your hips and with your eyes closed. You should try to maintain stability in that position for 20 seconds. I will be counting the number of times you move out of this position. I will start timing when you are set and have closed your eyes."

(b) Single leg stance:

"If you were to kick a ball, which foot would you use? [This will be the dominant foot] *Now stand on your non-dominant foot. The dominant leg should be held in approximately 30 degrees of hip flexion and 45 degrees of knee flexion. Again, you should try to maintain stability for 20 seconds with your hands on your hips and your eyes closed. I will be counting the number of times you move out of this position. If you stumble out of this position, open your eyes and return to the start position and continue balancing. I will start timing when you are set and have closed your eyes."*

(c) Tandem stance:

*"Now stand heel-to-toe with your **non-dominant foot** in back. Your weight should be evenly distributed across both feet. Again, you should try to maintain stability for 20 seconds with your hands on your hips and your eyes closed. I will be counting the number of times you move out of this position. If you stumble out of this position, open your eyes and return to the start position and continue balancing. I will start timing when you are set and have closed your eyes."*

Balance testing – types of errors

1. Hands lifted off iliac crest
2. Opening eyes
3. Step, stumble, or fall
4. Moving hip into > 30 degrees abduction
5. Lifting forefoot or heel
6. Remaining out of test position > 5 sec

Each of the 20-second trials is scored by counting the errors, or deviations from the proper stance, accumulated by the athlete. The examiner will begin counting errors only after the individual has assumed the proper start position. **The modified BESS is calculated by adding one error point for each error during the three 20-second tests. The maximum total number of errors for any single condition is 10.** If a athlete commits multiple errors simultaneously, only one error is recorded but the athlete should quickly return to the testing position, and counting should resume once subject is set. Subjects that are unable to maintain the testing procedure for a minimum of **five seconds** at the start are assigned the highest possible score, ten, for that testing condition.

Which foot was tested: ☐ Left ☐ Right
(i.e. which is the **non-dominant** foot)

Condition	Total errors
Double Leg Stance (feet together)	of 10
Single leg stance (non-dominant foot)	of 10
Tandem stance (non-dominant foot at back)	of 10
**Balance examination score** (30 **minus** total errors)	of 30

7 Coordination examination

Upper limb coordination

Finger-to-nose (FTN) task: *"I am going to test your coordination now. Please sit comfortably on the chair with your eyes open and your arm (either right or left) outstretched (shoulder flexed to 90 degrees and elbow and fingers extended). When I give a start signal, I would like you to perform five successive finger to nose repetitions using your index finger to touch the tip of the nose as quickly and as accurately as possible."*

Which arm was tested: ☐ Left ☐ Right

Scoring: 5 correct repetitions in < 4 seconds = 1

Note for testers: Athletes fail the test if they do not touch their nose, do not fully extend their elbow or do not perform five repetitions. Failure should be scored as 0.

Coordination score of 1

8 Cognitive assessment

Standardized Assessment of Concussion (SAC)

Delayed recall

"Do you remember that list of words I read a few times earlier? Tell me as many words from the list as you can remember in any order."

Circle each word correctly recalled. Total score equals number of words recalled.

List	Alternative word list		
elbow	candle	baby	finger
apple	paper	monkey	penny
carpet	sugar	perfume	blanket
saddle	sandwich	sunset	lemon
bubble	wagon	iron	insect

Delayed recall score of 5

Overall score

Test domain	Score
Symptom score	of 22
Physical signs score	of 2
Glasgow Coma score (E + V + M)	of 15
Balance examination score	of 30
Coordination score	of 1
**Subtotal**	**of 70**
Orientation score	of 5
Immediate memory score	of 5
Concentration score	of 15
Delayed recall score	of 5
**SAC subtotal**	**of 30**
**SCAT2 total**	**of 100**
**Maddocks Score**	**of 5**

Definitive normative data for a SCAT2 "cut-off" score is not available at this time and will be developed in prospective studies. Embedded within the SCAT2 is the SAC score that can be utilized separately in concussion management. The scoring system also takes on particular clinical significance during serial assessment where it can be used to document either a decline or an improvement in neurological functioning.

Scoring data from the SCAT2 or SAC should not be used as a stand alone method to diagnose concussion, measure recovery or make decisions about an athlete's readiness to return to competition after concussion.

Athlete Information

Any athlete suspected of having a concussion should be removed from play, and then seek medical evaluation.

Signs to watch for

Problems could arise over the first 24-48 hours. You should not be left alone and must go to a hospital at once if you:

- Have a headache that gets worse
- Are very drowsy or can't be awakened (woken up)
- Can't recognize people or places
- Have repeated vomiting
- Behave unusually or seem confused; are very irritable
- Have seizures (arms and legs jerk uncontrollably)
- Have weak or numb arms or legs
- Are unsteady on your feet; have slurred speech

Remember, it is better to be safe.
Consult your doctor after a suspected concussion.

Return to play

Athletes should not be returned to play the same day of injury. When returning athletes to play, they should follow a stepwise symptom-limited program, with stages of progression. For example:

1. rest until asymptomatic (physical and mental rest)
2. light aerobic exercise (e.g. stationary cycle)
3. sport-specific exercise
4. non-contact training drills (start light resistance training)
5. full contact training after medical clearance
6. return to competition (game play)

There should be approximately 24 hours (or longer) for each stage and the athlete should return to stage 1 if symptoms recur. Resistance training should only be added in the later stages.
Medical clearance should be given before return to play.

Tool	Test domain	Time	Score			
		Date tested				
		Days post injury				
**SCAT2**	Symptom score					
	Physical signs score					
	Glasgow Coma score (E + V + M)					
	Balance examination score					
	Coordination score					
**SAC**	Orientation score					
	Immediate memory score					
	Concentration score					
	Delayed recall score					
	**SAC Score**					
**Total**	**SCAT2**					
**Symptom severity score (max possible 132)**						
**Return to play**			☐ Y ☐ N	☐ Y ☐ N	☐ Y ☐ N	☐ Y ☐ N

Additional comments

Concussion injury advice (To be given to concussed athlete)

This patient has received an injury to the head. A careful medical examination has been carried out and no sign of any serious complications has been found. It is expected that recovery will be rapid, but the patient will need monitoring for a further period by a responsible adult. Your treating physician will provide guidance as to this timeframe.

If you notice any change in behaviour, vomiting, dizziness, worsening headache, double vision or excessive drowsiness, please telephone the clinic or the nearest hospital emergency department immediately.

Other important points:

- **Rest and avoid strenuous activity for at least 24 hours**
- **No alcohol**
- **No sleeping tablets**
- **Use paracetamol or codeine for headache. Do not use aspirin or anti-inflammatory medication**
- **Do not drive until medically cleared**
- **Do not train or play sport until medically cleared**

Clinic phone number

Patient's name

Date/time of injury

Date/time of medical review

Treating physician

Contact details or stamp
